# Total ammonia and coliform concentrations at the end of the Mississippi River from 1900 to 2019

**DOI:** 10.1007/s10661-022-10903-1

**Published:** 2023-01-07

**Authors:** R. Eugene Turner

**Affiliations:** Department of Oceanography and Coastal Sciences, Louisiana State University, Baton Rouge, LA 70803 USA

**Keywords:** Ammonia, Coliforms, Mississippi River, Clean water Act, Long term

## Abstract

Total ammonia (TA) concentrations (NH_3 +_ NH_4_^+^) at four locations at the terminal end of the Mississippi River, the largest river on the North American continent, were assembled to examine trends and relationships with point and non-point loadings from 1980 to 2019 and compared to values in 1900 to 1901. TA concentrations were lowest in 1900 to 1901, highest in 1980 and then declined, and then rose slightly in the last 2 decades. Variations in individual measurements and in situ temperature are indirectly related because of the influence temperature has on ammonia solubility and protein degradation rates. Importantly, the average annual concentrations of TA were directly related to both total coliform and fecal coliform densities. The highest measured average annual TA concentrations in the river (15.5 ± 1.5 SE µmol in 1985) were below the currently recommended toxicity thresholds for freshwater aquatic ecosystems. Sewerage loadings are implicated as controlling factors on TA concentrations, not nitrogen stabilizers added to fertilizers to reduce ammonia conversion to nitrate, nor the fertilizer loadings.

## Introduction

The Mississippi River watershed, the largest in North America, underwent striking changes in both point and non-point source loadings over the last two centuries as the principally European-based colonization converted forests and grassland habitats to farm fields and discharged sewerage and industrial pollutants. These changes diminished water quality as it has elsewhere (Meybeck, 2003), which was reversed in some instances after implementing the Clean Air Act (1963), Clean Water Act (1972), and other national legislation in the 1970s. Average oxygen concentrations at the river’s end in New Orleans, for example, increased by 0.21 mg dissolved oxygen decade^−1^ from 1967 to 2019, and the monthly pH in the river increased from a low of 5.8 in 1965 to 8.2 in 2019. Total coliform densities, fecal coliform densities, and lead concentrations declined several orders of magnitude over the same interval—principally because of point source controls on sewage and air pollution (Turner, 2021). In contrast, non-point sources in the upper watershed resulted in nitrate concentrations in the lower river doubling since the early 1900s, and both silicate concentrations and suspended sediments declined by 50% as a result of land use changes (Meade & Moody, 2010; Mize et al., 2018; Turner & Rabalais, 2003).

The 58% of the watershed that is cropland today produces about one-third of the world’s corn and soybean (Food and Agriculture Organization of the United Nations, 2021) and used 65% of the US fertilizer applications in 2014, equivalent to 8.1 TgN y^−1^ (Tian et al., 2020). The average N fertilizer use rate in the USA increased from less than 0.01 gN m^−2^ yr^−1^ in 1850 to 9.04 g N m^−2^ yr^−1^ in 2015 and was > 6 gN m^−2^ yr^−1^ in the watershed by 2015 (Cao et al., 2018) and declined only 1.58% during 2002–2012 (Crawford et al., 2019). This fertilizer is a non-point source of nitrogen and may contribute significant amounts of TA to the river’s terminal end.

The dry form of the fertilizer applied is comprised of ion ammonium (NH_4_^+^) or urea, and the wet form is unionized ammonia (NH_3_; Cao et al., 2018). Ammonia (NH_3_) in water forms ammonium (NH_4_^+^) and hydroxyl (OH^−^) ions:

1 $${\mathrm{NH}}_{3}+{\mathrm{H}}_{2}\mathrm{O }\leftrightarrow {\mathrm{NH}}_{4}\mathrm{OH }\leftrightarrow {{\mathrm{NH}}_{4}}^{+}+{\mathrm{OH}}^{-}$$

A pH above 7.2 favors NH_3_, and this increases with a higher pH. The sum of the ionized ammonia (NH_4_^+^) and unionized ammonia (NH_3_) is the total ammonia (TA) and is what is usually measured in water samples. The oxidation of TA is a two-step process involving aerobic chemoautotrophic organisms that results in the formation of nitrite and nitrate. Non-point runoff, sourced mostly from improvements in field drainage, contains high amounts of nitrate that are derived from NH_3_ and NH_4_^+^ (David et al., 2010; Jones & Schilling, 2013). This nitrogen fertilizer leaking from croplands as nitrate has become a significant controlling factor on the size of the hypoxic (< 2 mg oxygen L^−1^) northern Gulf of Mexico coast, which is the second largest human-caused coastal hypoxic area in the global ocean (Rabalais & Turner, 2019). Feedlots produce additional TA as both NH_3_ volatilizations and runoff (Jones et al., 2019).

Nitrogen stabilizers co-applied to fertilizers may affect how much of this TA enters waterways. Stabilizers in the USA contain nitripyrin (Woodward et al., 2021) that was first registered with the Environmental Protection Agency in 1974; 99% of nitripyrin is used on corn (U.S. Environmental Protection Agency, 2005) in 24% of the N fertilizer applications in the USA (Woodward et al., 2021). It is promoted as a bactericide that retards or stops the conversion of ammonium to nitrite, or nitrite to nitrate. Reducing nitrification might indicate that more ammonium is available for root uptake and that nitrate movements off-field are reduced, but also that TA export increased. A recent review concluded that nitrate leaching may be increased or decreased with nitripyrin use and that NH_3_ volatilization increases from 3 to 150% (Woodward et al., 2021). TA concentrations in the river are, therefore, influenced by point sources (sewerage) and potentially non-point (agricultural) inputs.

Here, I compare trends represented in three differently sourced water quality data sets containing TA concentrations in water at the terminal end of the Mississippi River. These data began in 1901 but are principally based on 1980 to 2019 water quality sampling programs. Annual TA concentrations are hypothesized to be proportional to bacterial concentrations entering from non-point sewerage sources that have been declining since the 1960s. The relative influence of point and non-point sources on ammonia concentrations is examined within this context.

## Methods

### Total ammonia concentrations

**Fig. 1:**
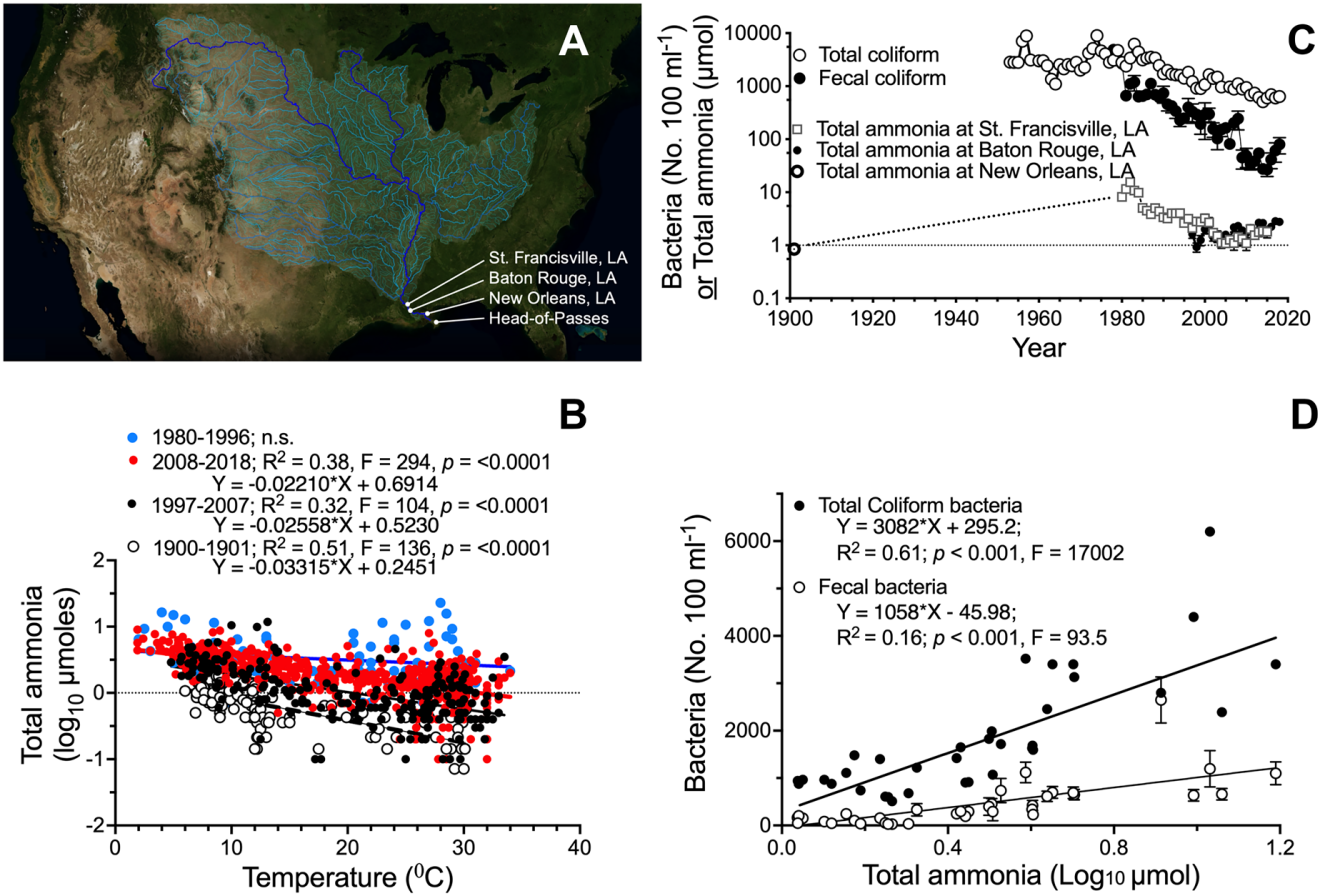
Total ammonia concentration relationships with temperature, years and bacteria concentrations. **A** Data collection locations at the end of the Mississippi River watershed and their position relative to the Head-of-Passes at the river’s terminus (map adapted from NASA’s Scientific Visualization Studio (https://svs.gsfc.nasa.gov/4493)). **B** The relationship between in situ temperature and Total ammonia concentrations at New Orleans in 1900−1901 (open circles), at Baton Rouge, Louisiana, from  1997−2007 and 2008−2018 (black and red circles, respectively), and at St. Francisville from 1980−1996 (blue circles). **C** The average annual densities of Total coliform bacteria and Fecal coliform bacteria at New Orleans, and average annual Total ammonia concentrations at St. Francisville and Baton Rouge. **D** The relationship between the average annual Total ammonia concentration at St. Francisville, Louisiana, and annual Total coliform and Fecal coliform densities at New Orleans, Louisiana. The mean ± 1 SE is shown

Data are from samples collected at three locations on the main stem of the Mississippi River (Fig. 1; Table 1). The United States Geological Survey (USGS, 2020) sampled at St. Francisville, LA, USA, located 428 river km upstream from the Head of Passes where the river divides into three main outlets to the Gulf of Mexico (Fig. 1A). The samples from Baton Rouge, LA, USA, 370 river km from the Head of Passes, were collected at least once monthly, and up to five times per month during spring and summer, from 1997 to 2018 (22 to 46 samples y^−1^). The data are in Turner et al. (2022); this data set also includes other water quality measurements on concentrations of inorganics, suspended sediments, total nitrogen, phosphorus, and carbon, among others. The calibration curves followed good laboratory practices and used automated analytical methods. The third water sampling locations are in the metropolitan area of New Orleans, LA, USA. One is from the river water intake pipe at the New Orleans Sewerage and Water Board (NOSWB) Carrollton water treatment plant 167 km from the Head of Passes. The same water intake location has been used continuously since it was first put in use in December 1900 ([NOWSB] Sewerage and Water Board, New Orleans, La, 1903). TA concentrations as well as the temperature at the time of sampling were determined in 155 samples collected from December 1900 to August 1901 ([NOWSB] Sewerage and Water Board, New Orleans, La, 1903). Not all sample collections had water temperatures measured.

**Table 1:** Data collection location, sample years, sample frequency, and data sources

Location	River mile	Years	Sample frequency	Data source
A. Total ammonia concentration				
St. Francisville, LA	428	1980–2016	12 mo^−1^	USGS (2020)
Baton Rouge, LA	270	1997–2018	2 to 4 mo^−1^	Turner et al. (2022)
Carrollton, LA	167	1900–1901	15 mo^−1^	NOWSB (1903)
B. Total coliform density				
Carrollton, LA	167	1968–2018	11 to 30 mo^−1^	Turner (2021)
C. Fecal coliform density				
Belle Chase, LA	122	1968–2018	0.8 to 2 mo^−1^	Turner (2021)

### Coliform bacteria

The total coliform bacteria concentrations are from water samples from the river water intake pipes at the NOSWB Carrollton water treatment plant. Estimates of the total coliform bacteria density in this water were reported annually from 1968 to 2018 (*n* = 139 to 382 y^−1^), but individual measurements in all years are no longer available, only the annual average. Fecal coliform densities in Mississippi River surface waters at Belle Chase, LA, USA (river mile 122) were measured by the LaDEQ (2020) using a membrane filtration method. The fecal coliform data were summarized by month and the median value (µ ± 1 SE) for the 12 months compared from 1968 to 2018. The two bacteria data sets are in the Supplemental Materials within Turner (2021). The water collection data on bacteria densities in New Orleans and the TA concentrations at St. Francisville and Baton Rouge were not often on the same sampling days.

### Statistics

I used Prism 8.0c software © 2020 (GraphPad Software, Inc., La Jolla, CA, USA) for statistical analyses where the significance is *p* < 0.05. A log_10_ transformation of the TA values versus temperature was plotted, and the simple linear regression between the data sets was tested for differences in regression slopes and their intercepts and also to compute an annual average and standard error (µ ± 1 SE). The annual average used here is the mean ± 1 SE for the 12 individual months. Log transforms were made for graphing purposes. A simple linear regression was done to test if there was a significant relationship between the TA concentration at St. Francisville and the fecal coliform concentrations at New Orleans.

## Results

### Total ammonia and temperature

TA concentrations were negatively related to the temperature at New Orleans and Baton Rouge (Fig. 1B). The slopes for the two intervals from 1997 to 2007 and 2008 to 2018 were not significantly different from each other, but the intercepts were different (*p* < 0.001, *F* = 116). The slopes of the latest interval (2008–2018) and the earliest (1900 to 1901) were also different from each other (*p* < 0.001, *F* = 11.2). The slopes for the two intervals from 1997 to 2008 and the earliest interval (1900–1901) were not different, but the intercepts were different (*p* < 0.0001, *F* = 133). In summary, TA concentrations in all three intervals had a negative relationship with temperature, and there were higher TA concentrations from 1997 to 2018 than from 1900 to 1901, and a slightly higher average concentration from 2008 to 2018 compared to 1997 to 2008.

### Bacteria and total ammonia

The TA concentrations at Baton Rouge and St. Francisville were of similar values for the same year; the average of all 19 years of overlapping data was 1.80 ± 0.15 and 1.80 ± 0.11 µmol at St. Francisville and Baton Rouge, respectively. The average TA concentration at New Orleans was 0.845 ± 0.042 µmol in 1901, 15.476 ± 1.517 µmol at St. Francisville in 1982, and then declined to 1.092 ± 0.296 µmol by 2010, before rising again to an average of 1.80 ± 0.08 µmol from 2011 to 2015 (Fig. 1C) when it was twice the concentration in 1901 at New Orleans, and 12% of the peak concentration in 1982 at St. Francisville. Note the rising TA values from 2000 to 2018 which was 0.06 µmol y^−1^ (3.1% y^−1^).

The average annual density of both total coliform and fecal coliform bacteria declined coincidentally as the average annual TA concentration became lower (Fig. 1C). The minimum total coliform densities in 2021 were about one-tenth the maximum concentrations measured between the 1960s and the 1980s (Fig. 1C). A linear regression of the annual average total coliform bacteria densities at New Orleans and TA at St. Francisville,was significant (*R*^2^ = 0.61, *p* < 0.001), but the correlation coefficient with fecal coliform bacteria was less so (*R*^2^ = 0.l6, *p* < 0.001; Fig. 1D). The TA concentration at St. Francisville and the fecal coliform density in New Orleans were significantly related in a simple linear regression equation where TA concentration = 0.08862*fecal concentration (*R*^2^ = 0.81; *F* = 150.4, *n* = 37, *p* < 0.001).

## Discussion

The average annual TA concentration at the lower end of the Mississippi River was 30 times higher in 1980 compared to the early 1900s as the watershed was being colonized. TA concentrations may have been higher before 1980, although the likely driving force, sewerage, appears to have been stabilized in the previous decades as indicated by the total coliform bacteria concentrations in the river. Both the total coliform and TA concentrations declined from about 1980 to 2010 and rose slightly after 2000. TA concentrations increased proportionally with total coliform bacteria and fecal coliform bacteria concentrations. Although this is a gross evaluation of TA concentrations at the bottom of the watershed, it supports the hypothesis that point source reductions in sewerage loading as the implementation of legislative mandates successfully resulted in a water quality improvement at the scale of the largest river in the USA.

The individual measurements of TA concentrations within broad temporal intervals varied with temperature, being highest in colder water. This relationship illustrates the greater solubility of TA and lower volatilization with declining temperature, and higher decomposition rates creating more TA at higher temperatures. The overlapping concentrations of average annual TA concentrations at stations 58 km downstream suggest that NH_3_ volatilization is less important than the loading of TA.

The TA concentrations in the river are declining despite the use of nitrogen stabilizers that presumably increase the conservation of TA in soil water and have the potential for drainage into waterways. Perhaps, the increased availability of soil water ammonium created is taken up by plants, as intended, volatilized in the farm field, or is inconsequential. Regardless, the hypothesis that nitrogen stabilizers are a significant influence on TA concentrations in the river is not supported by this analysis.

The percentage of the TA that is in the unionized form (NH_3_) increases with higher temperature and pH (Emerson et al., 1975). This unionized form has toxic effects on invertebrates and fish (Fan et al., 2021), which have led to various acute and chronic aquatic life criteria, commonly referenced to a pH of 7.0 and 20 °C. Park et al. (2018) based their species sensitivity criteria for Korean indigenous aquatic biota when they estimated that the hazardous concentration for 5% of biological species was 1751 µmol. The TA criteria for acute and chronic TA in the USA are 1214 and 136 µmol, for acute exposure over 24 h and chronic exposure over 30 days, respectively (USEPA, 2013). Zhang et al. (2018) estimated acute criteria of 81 µmol for TA for 98 to 145 annual samples taken from 2007 to 2014 in China. These acute and chronic thresholds are far higher than the 15 µmol annual average TA observed in the Mississippi River.

## Conclusions

The concentration of total ammonia (NH_3_ + NH_4_^+^) in the Mississippi River increased an order of magnitude from 1900/01 to 1980 but declined after the Clean Air Act, Clean Water Act, and related legislative initiatives, after which the concentration of TA tracked the dramatic declines in bacteria concentration. Following the declines, there was a slight rise in both after 2000 of 3.1% y^−1^. Individual measurements of TA concentration in various intervals were inversely related to temperature. The TA concentrations never exceeded water quality guidelines/criteria for TA intended to protect species. The TA concentrations are not influenced by agricultural non-point sources because of soil nitrogen stabilizers containing nitripyrin, but rather by point sources.

## Data Availability

The datasets analyzed during the current study are available at the USGS website (http://toxics.usgs.gov/hypoxia/mississippi/flux_ests/delivery/index.html), the Dryad repository (https://doi.org/10.5061/dryad.x95x69pkm), the Louisiana Department of Environmental Quality website (https://waterdata.deq.louisiana.gov/), and in Turner (2021) and Sewerage and Water Board (1903) that are cited in this manuscript.
